# Eukaryotes may play an important ecological role in the gut microbiome of Graves’ disease

**DOI:** 10.3389/fimmu.2024.1334158

**Published:** 2024-02-22

**Authors:** Xiwen Geng, Yalei Liu, Wenbo Xu, Gefei Li, Binghua Xue, Yu Feng, Shasha Tang, Wei Wei, Huijuan Yuan

**Affiliations:** ^1^ Department of the Clinical Research Center, Henan Provincial People’s Hospital, People’s Hospital of Zhengzhou University, Zhengzhou, Henan, China; ^2^ Department of Endocrinology, Henan Provincial Key Medicine Laboratory of Intestinal Microecology and Diabetes, Henan Provincial People’s Hospital, People’s Hospital of Zhengzhou University, Zhengzhou, Henan, China; ^3^ Department of Clinical Microbiology, Henan Provincial People’s Hospital, People’s Hospital of Zhengzhou University, Zhengzhou, Henan, China; ^4^ Department of Blood Transfusion, Henan Provincial People’s Hospital, Department of Blood Transfusion of Central China Fuwai Hospital, Central China Fuwai Hospital of Zhengzhou University, Zhengzhou, Henan, China

**Keywords:** gut microbiome, multi kingdom microbiome, community assembly, hyperthyroidism, inflammatory factors

## Abstract

The prevalence of autoimmune diseases worldwide has risen rapidly over the past few decades. Increasing evidence has linked gut dysbiosis to the onset of various autoimmune diseases. Thanks to the significant advancements in high-throughput sequencing technology, the number of gut microbiome studies has increased. However, they have primarily focused on bacteria, so our understanding of the role and significance of eukaryotic microbes in the human gut microbial ecosystem remains quite limited. Here, we selected Graves’ disease (GD) as an autoimmune disease model and investigated the gut multi-kingdom (bacteria, fungi, and protists) microbial communities from the health control, diseased, and medication-treated recovered patients. The results showed that physiological changes in GD increased homogenizing dispersal processes for bacterial community assembly and increased homogeneous selection processes for eukaryotic community assembly. The recovered patients vs. healthy controls had similar bacterial and protistan, but not fungal, community assembly processes. Additionally, eukaryotes (fungi and protists) may play a more significant role in gut ecosystem functions than bacteria. Overall, this study gives brief insights into the potential contributions of eukaryotes to gut and immune homeostasis in humans and their potential influence in relation to therapeutic interventions.

## 1 Introduction

The vital role of the gut microbiome in immune homeostasis and the maintenance of health in humans has been highlighted in numerous studies, revealing that the gut microbiome is inextricably linked to the development of various metabolic and autoimmune diseases. Graves’ disease (GD) is a common autoimmune disease and a major cause of hyperthyroidism. It occurs at all ages and is more common in women (1). Previous research mainly focused on the disruption of microbial taxa in GD patients, involving increased *Prevotellaceae*, *Lactobacillales*, and *Bacilli* and decreased *Rikenellaceae*, *Alistipes*, and *Enterobacteriaceae* (2, 3). However, previous studies only focused on the intestinal bacterial communities, while eukaryotes (such as fungi and protists) have rarely been studied. Nevertheless, the human gut microbiome is composed of multi-kingdom microbial communities that play indispensable but largely unrecognized roles. Turning our attention to the “microbial zoo” of the gut ecosystem will help to reveal the mechanisms underlying the link between the microbiome and immune disorders (4, 5).

The assembly and succession of complex gut microbiomes have attracted much attention in recent years. The assembly of ecological communities is normally influenced by both stochastic and deterministic processes (6). Deterministic processes indicate that the community diversity and structure are directional and predictable (7). For instance, diet (8), host genetics (9), medication use (10), and endocrine factors (11) can partially explain inter-individual microbiome variation via deterministic processes, while the external environmental factors seem impossible to influence communities through stochastic processes (also known as neutral processes), such as dispersal, specialization, and drift, even though they may also play a role in ecosystem shaping (12). In various health and disease states, the microbial load itself may be an identifier of a particular ecosystem configuration (13). However, the microbiome assembly processes in recovered patients after intervention, as well as the improvement of their gut dysbiosis, are generally overlooked. Furthermore, although interactions between certain physiological drivers and microbiome assembly have been studied (4, 14), our understanding of the ecological mechanisms underlying disordered and recovered gut microbiomes remains vague. Understanding these mechanisms is crucial for understanding overall health outcomes of GD.

In this study, we collected samples from 59 individuals, comprising 20 GD patients, 19 GD patients who had recovered after taking medication, and 20 healthy people, and assessed the ecological processes underlying their microbial kingdom (bacterial, fungal, and protistan) community assembly. In addition, we performed inter-kingdom analysis within each group to further explore the interactions among the microbial kingdoms and physiological indexes. The results extend our knowledge of GD in terms of the multi-kingdom microbial ecology, and provide a potential direction for the development of novel GD treatments based on gut fungi and protists as they may play a more significant role in gut ecosystem functions than bacteria.

## 2 Materials and methods

### 2.1 Participant recruitment and sample collection

From March 2020 to March 2021, 59 participants, comprising (1) 20 GD patients (Disease group), (2) 19 GD patients who recovered after medication treatment (Recovered group), and (3) 20 healthy controls (Healthy group), were recruited from the central plains of China. The diagnostic criteria for GD and the inclusion and exclusion criteria for the participants are described in detail in the **Supplementary Materials**.

All participants fasted overnight (≥8 h) before sample collection. Fecal samples were collected for DNA extraction, and serum samples were collected to assess thyroid hormones and inflammatory factors.

### 2.2 Measurement of physiological indexes

Participants’ demographic and clinical data were collected using questionnaires and electronic medical records. Chemiluminescence immunoassays were performed to assess free tetraiodothyronine (FT4), free triiodothyronine (FT3), thyroid-stimulating hormone (TSH), and thyrotropin receptor auto-antibodies (TRAb) using a Cobas e602 analyzer (Roche Diagnostics, Switzerland), and to assess thyroid peroxidase antibodies (TPOAb) and anti-thyroglobulin antibodies (TgAb) using a UniCel DxI 800 analyzer (Beckman Coulter, USA). Serum levels of inflammatory factors (tumor necrosis factor α, TNFα; interleukin 4, IL4; interleukin 6, IL-6; interleukin 10, IL10; interleukin 17, IL17) were assessed using human enzyme-linked immunosorbent assay (ELISA) kits (Cusabio Biotech, Wuhan, China). Detailed information is provided in **Supplementary Table S1**.

### 2.3 DNA extraction and Illumina MiSeq sequencing

The total DNA was extracted from the fecal samples using a QIAamp Fast DNA Stool Mini Kit (QIAGEN, Germany). DNA concentration and quality were assessed using a NanoDrop 2000 spectrophotometer (Thermo Fisher, USA). To amplify the V5–V7 region of the bacterial 16S rRNA gene, the ITS1 region of the fungal ITS gene, and the V4 region of the protistan 18S rRNA gene, the following primer pairs were used: 799F/1193R (799F: 5′-AAC MGG ATT AGA TAC CCK-3′; 1193R: 5′-ACG TCA TCC CCA CCT TCC-3′) (15), ITS1F/ITS2 (ITS1F: 5′-CTT GGT CAT TTA GAG GAA GTA A-3′; ITS2: 5′-GCT GCG TTC TTC ATC GAT GC-3′) (16, 17), and V4_1F/TAReukREV (5′-CCA GCA SCY GCG GTA ATW CC-3′; 5′-ACT TTC GTT CTT GAT YRA-3′) (18), respectively. Amplicon sequencing was conducted using an Illumina MiSeq platform by Magigen Biotechnology Co., Ltd. (Guangzhou, China).

Quality control of the sequence reads was performed using the UPARSE pipeline (19). Paired-end reads were assembled and trimmed (maximal expected errors of 0.25, reads length >300 bp for bacteria and protists, reads length >200 bp for fungi). Filtered sequences were clustered into 100% sequence similar zero-radius operational taxonomic unit (zOTU) using the UNOISE 3 algorithm implemented in USEARCH. For the bacterial communities, this step generated a 16S zOTU table of 59 samples × 3,370 zOTUs (3,275,695 reads). The number of high-quality sequences per sample was 31,660–95,736. For the fungal communities, this step generated an ITS zOTU table of 51 samples × 3,006 zOTUs (2,528,186 reads, the sample with high-quality sequences less than 3,000 was discarded). The number of high-quality sequences per sample was 3,615–133,846. Bacterial and fungal zOTUs were classified by the RDP classifier against the RDP 16 S rRNA gene database and the UNITE ITS database, respectively (20). Eukaryotic zOTUs were classified against the Protist Ribosomal Reference database (PR2) (21). We discarded zOTUs assigned as Rhodophyta, Streptophyta, Metazoa, Fungi, and unclassified Opisthokonta sequences to obtain the protistan zOTU table. For the protistan communities, we finally obtained a zOTUs table of 50 samples × 5,800 zOTUs (1,043,313 reads, the sample with high-quality sequences less than 3,000 was discarded). The number of high-quality sequences per sample was 3,062–61,354. To obtain an equivalent sequencing depth for further bioinformatics analysis, each sample was rarefied to 31,660 for bacterial communities, 3,615 sequences for fungal communities, and 3,062 for protistan communities in R through the package “GUniFrac” (Function: Rarefy).

### 2.4 Bioinformatics analysis of microbial sequencing results

Alpha diversity indicators, that is, richness (total number of observed species [Sobs]) and Shannon index, were determined for each sample using “vegan” (function: diversity) in R v4.2.2 for Windows. The differences among groups were determined based on the Wilcoxon signed-rank test using “ggpubr”.

The Bray–Curtis distance among different groups was determined using “vegan” based on zOTUs table, and the dissimilarities in microbial community composition were then visualized based on principal coordinate analysis (PCoA) plots using “ggplot2”. Subsequently, permutational multivariate analysis of variance (PERMANOVA) was conducted using “vegan” (function: adonis) with 9,999 permutations to assess the dissimilarities in community structure among different groups. The differences among groups were determined using Kruskal-Wallis test in IBM SPSS version 26.

The major ecological processes were determined in order to disentangle the characteristics of gut microbial community assembly for each kingdom (bacteria, fungi, and protists) in the Disease, Recovered, and Healthy groups. First, a neutral community model (NCM) was applied to predict the potential importance of stochastic processes in community assembly by determining the relationships between the microbial taxa detection frequency in a group of communities and their relative abundance across the metacommunity of all groups (22). Then, the dynamics of phylogenetic and taxonomic diversity were assessed using beta nearest taxon indices (βNTI) based on the null-model and Bray–Curtis-based Raup–Crick (RC_Bray_) metrics through the R package “iCAMP”. The value of |βNTI| > 2 indicates that the deterministic processes, which can be divided into homogeneous selection (βNTI < −2, leading to similar community structures in similar environments) and variable selection (βNTI > 2, leading to dissimilar community structures in heterogeneous conditions), primarily govern community assembly. Conversely, |βNTI| < 2 suggests that stochastic processes govern the community compositions. Then, the RC_Bray_ was used to partition the stochastic processes. |RC_bray_| > 0.95 indicates homogenizing dispersal (RC_bray_ < −0.95) or dispersal limitation (RC_bray_ > 0.95) drives compositional turnover. When |βNTI| < 2 and |RC_bray_| < 0.95, this estimates the influence of “undominated” assembly, including weak dispersal, weak selection, diversification, and/or drift (23). The Linear regression model between physiological indexes and βNTI was calculated through the R package “ggpmisc”.

To evaluate the differences among all groups, an inter-kingdom network was separately constructed for each group. The zOTU tables were firstly calculated to the taxa table on a genus level. Then, the taxa tables for each dataset were limited to taxa present in at least half of the samples and comprised taxa with relative abundance ≥0.1% in each group. For each dataset, Spearman correlation scores were calculated in the MENA online pipeline (http://ieg4.rccc.ou.edu/mena/). The inter-kingdom co-occurrence networks were visualized in Gephi (version 0.9.2 for Windows).

## 3 Results

There were no significant differences in age or body mass index (BMI) among the three groups. Regarding the thyroid hormones, FT3 and FT4 were significantly lower in the Healthy and Recovered groups than the Disease group. TSH, TgAb, TPOAb, and TRAb were similar between the Disease and Recovered groups, but there were significant differences compared to the Healthy group. There were no significant differences in inflammatory factors among the three groups (**Supplementary Figure S1**).

**Figure 1:**
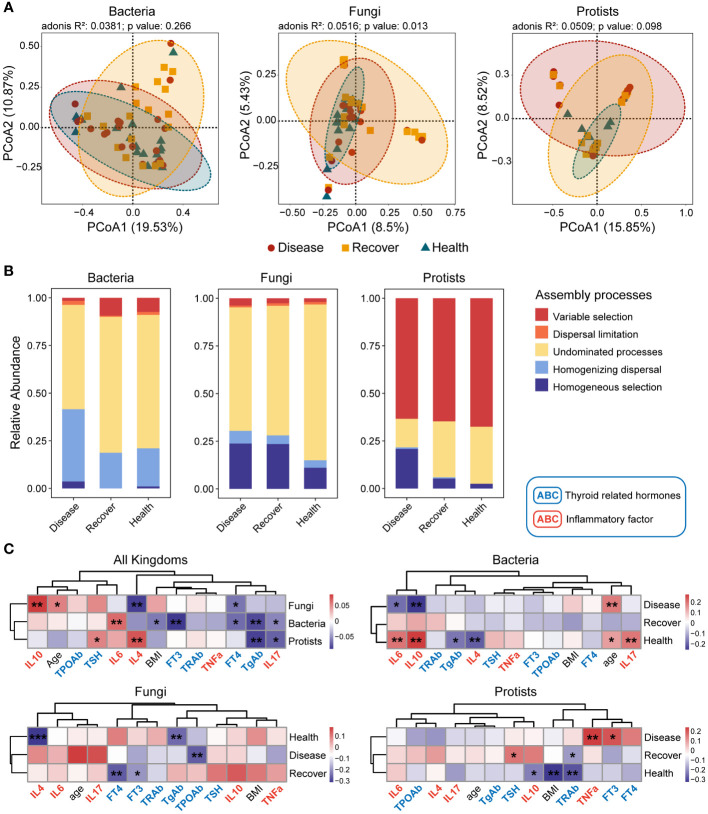
Bacterial, fungal, and protistan community compositions and their assembly processes. **(A)** PCoA plots based on Bray–Curtis distance for bacterial, fungal, and protistan communities. The *p*-values were calculated using PERMANOVA. **(B)** Relative influences of distinct processes on microbial community assembly. **(C)** Correlations between βNTI values and differences in physiological indexes. *p ≤ 0.05, **p ≤ 0.01 based on linear regression.

The sequencing results of the bacterial, fungal, and protistan communities revealed that there were no significant differences in alpha diversity (Sobs or Shannon index) among the three groups (**Supplementary Figure S2**). There were differences among the three groups in the bacterial, fungal, and protistan communities based on Bray–Curtis distance, but the differences were only significant for the fungal communities (**Figure 1A**).

The three kingdoms all exhibited more convergence in the PCoA plots in the Healthy group. Therefore, we determined the assembly processes for each of the microbial communities. The NCM effectively predicted a significant portion of the associations between the frequency of occurrence of bacterial zOTUs and their changes in relative abundance, but it failed for fungal and protistan zOTUs. The NCM results indicated that stochastic processes were more important for bacterial community assembly in the Disease and Recovered groups than the Healthy group (**Supplementary Figures S3–S5**). We then evaluated the assembly processes based on the β-nearest taxon index (βNTI) and Bray–Curtis-based Raup–Crick (RC_Bray_). Regarding bacterial community assembly, we found that homogenizing dispersal and undominated processes were the dominant processes, but there was more variable selection in the Healthy and Recovered groups than the Disease group. Regarding fungal community assembly, undominated processes dominated, but there was more homogeneous selection in the Disease and Recovered groups than the Healthy group. Lastly, regarding protistan community assembly, variable selection dominated, and there were more homogeneous selection and less undominated processes in the Disease group than the Recovered and Healthy groups (**Supplementary Figure S6**; **Figure 1B**). Based on family- and genus-level taxonomy analysis, the bacterial and protistan communities between the Healthy and Recovered groups were found to be similar. However, in terms of the fungal community, similarities were observed between the Disease and Recovered groups (**Supplementary Figures S7, S8**). Specifically, Lachnospiraceae and Eggerthellaceae were significantly higher in Disease than other bacterial groups. The abundance of Saccharomycetales incertae sedis was significantly lower in Healthy compared to other fungal groups, while Piptocephalidaceae and Spizellomycetaceae were significantly higher in Healthy. Blastocystis was higher in Healthy and Recovered than that in the protistan group of Disease (**Supplementary Figure S7**).

We also evaluated the relationships between βNTI values and physiological indexes (thyroid hormones and inflammatory factors) to further analyze the processes’ relative influences regarding mediating microbial community assembly. Many physiological indexes (such as TSH, FT3, FT4, TgAb, IL4, IL6, IL10, and IL17) influenced the microbial community assembly. There was a similar assembly process, influenced by thyroid hormones and inflammatory factors, for bacterial and protistan communities but not fungal communities (**Figure 1C**).

Moreover, we analyzed the assembly processes for each microbial kingdom in depth. The results showed that physiological indexes in the Recovered vs. Healthy groups similarly influenced bacterial and protistan community assembly, and physiological indexes in the Disease vs. Recovered groups similarly influenced fungal community assembly. The results indicated that the three microbial kingdoms in the Disease vs. Healthy groups were assembled via different processes, and the Recovered group fell between these two.

**Figure 2:**
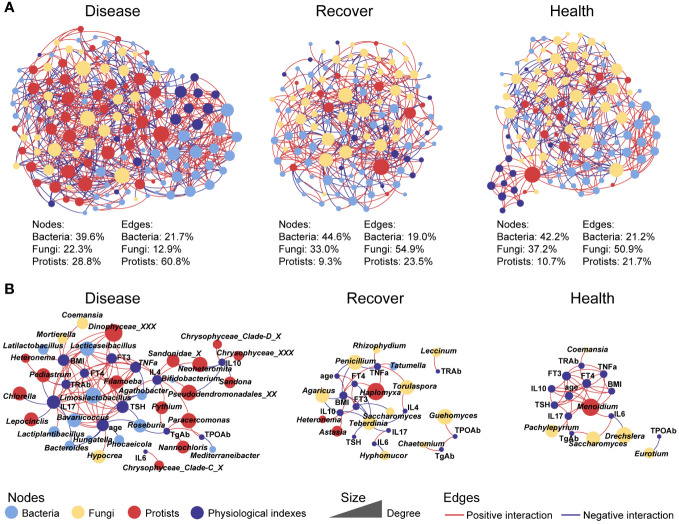
Co-occurrence networks between physiological indexes and all microbial kingdoms in each group. **(A)** Networks involving physiological indexes and all microbial taxa. **(B)** Networks involving physiological indexes and their associated microbial taxa.

To further explore the interactions among the microbial kingdoms and physiological indexes, we constructed separate inter-kingdom networks for the Disease group (139 nodes and 635 edges), Recovered group (118 nodes and 379 edges), and Healthy group (121 nodes, 387 edges). The Disease network was the most complicated. Interestingly, the protists in the Disease network had 28.8% of nodes that were related to 60.8% of edges, which were much higher values than in the Recovered network (9.3% of nodes and 23.5% of edges) and Healthy network (10.7% of nodes and 21.7% of edges). There were also relatively fewer fungal nodes and edges in the Disease network (22.3% of nodes and 12.9% of edges) than the Recovered (33.0% of nodes and 54.9% of edges) and Healthy (37.2% of nodes and 50.9% of edges) networks. Moreover, we constructed three sub-networks that were based on all the nodes directly associated with physiological indexes, and we found that there were more protists and bacteria in the Disease network than the Recovered and Healthy networks, indicating the consequence of the ecological assembly under GD, which involves the physiological changes caused by GD, such as IL17, TSH, and TNFa, complexed with the gut microbial inter-kingdom interaction. Specifically, edges associated with bacterial taxonomy, such as *Limosilactobacillus*, *Lacticaseibacillus*, *Bavariicoccus*, and *Roseburia*, and Protistan taxonomy, such as *Filamoeba*, *Paracercomonas*, *Pseudodendromonadales*_XX, and *Neoheteromita*, were increased in Disease. The edges associated with fungal taxonomy, such as *Torulaspora*, *Guehomyces*, *Saccharomyces*, and *Eurotium*, were increased in Recovered or Healthy (**Figure 2**).

## 4 Discussion

The thyroid gland influences the metabolic and immune processes in the body by producing thyroid hormones, which can lead to microbiome–thyroid homeostasis or disorder. This study explored the influence of physiological changes on gut microbial community assembly. We found that stochastic processes dominated the bacterial and fungal community assembly, while deterministic processes dominated the protistan community assembly. GD increased homogenizing dispersal processes for bacterial community assembly, and increased homogeneous selection processes for eukaryotic community assembly. This may be due to GD-induced changes in the gut environment that reduced the stability of the gut bacterial community and simultaneously altered the bacterial metabolism and reproduction rate, making it easier for fungi and protists to survive and reproduce under these specific environmental conditions (24–26). However, the medication-treated recovered patients vs. healthy controls had similar bacterial and protistan, but not fungal, community assembly processes. This concurs with the similarity of recovered patients vs. healthy controls in other disease models, although previous research mainly focused on bacterial communities (27, 28). The state of the fungal community in recovered patients may be related to the stability of this community (29). Gut bacteria are already regarded as specific diagnostic biomarkers for GD (30), and fungal and protistan biomarkers of disease progression should be identified in future research.

The functions of gut bacteria have been widely discussed in many studies (31, 32), while the functions of eukaryotes, which may explain many currently unexplained variables, have mostly been overlooked. In this study, fungi contributed more than half of the interactions among the multi-kingdom communities in the Healthy and Recovered groups. Disruption of the mycobiota can have detrimental impacts on the host immune system (33), and Leonardi et al. demonstrated that mucosal fungi in mice enhanced intestinal epithelial functions and protected against bacterial infection and intestinal injury (34). Specifically, *Torulaspora* and *Saccharomyces*, which belong to Saccharomycetales and *Eurotium*, contributed more interactions in Healthy and Recovered groups. Similarly, many reports have demonstrated that *Saccharomyces* could be used as a biotherapeutic agent owing to its anti-inflammatory, antibacterial, and immune modulatory properties (35, 36). *Eurotium* can also exhibit anti-colitis effects through regulating gut microbiota-dependent tryptophan metabolism (37). Therefore, fungi may play a vital role in maintaining health and avoiding GD in humans.

Additionally, protists dominated the interactions in multi-kingdom microbial communities of disease group. Predatory protozoa and algae contribute to these connections, which may be due to the disruption of the gut environment leading to an increase in bacteria, thereby enhancing the connection between protozoa and other microorganisms. Specific eukaryotic microbes in the gut ecosystem have been reported to have a role in modulating the host immune system (5). Over millions of years, eukaryotic microbes co-evolved with mammals; although there are fewer eukaryotic microbes than bacteria living in the gut, they are much larger in size and may have a disproportionate influence (5, 38). In this study, blastocystis was enriched in Healthy and Recovered groups. Blastocystis is a protistan parasite and also a common component of the healthy gut microbiome, and recent studies have reported that *Blastocystis* have a potentially beneficial effect on regulating host immune responses (39). Moreover, the latest report also emphasized the importance of commensal protists for regulating intestinal immunity and trans-kingdom competition (40). However, the role of protists in gut microbiome assembly in healthy humans remains mostly unrecognized. Thus, we argue that eukaryotic microbes, especially protists, represent an essential factor that should be taken into consideration when analyzing the gut microbiome.

Overall, this study highlights the intricate yet previously unexplored dynamics of gut multi-kingdom microbiome assembly in GD patients and medication-treated patients who recovered from GD. We conclude that, via physiological changes, GD increased homogenizing dispersal processes for bacterial community assembly and increased homogeneous selection processes for eukaryotic community assembly. The bacterial and protistan, but not fungal, assembly processes of the medication-treated recovered patients vs. healthy controls were similar. Furthermore, eukaryotic microbes potentially contributed more ecosystem functions than bacteria in the gut environment. This study gives brief insights into the potential contributions of eukaryotic microbes to gut and immune homeostasis in humans and their potential influence in relation to therapeutic interventions. Notably, more research into the potential benefits of eukaryotic microbes in humans will offer many exciting prospects for revealing the mechanisms of establishment and the complex traits of immune diseases.

## Data availability statement

Raw sequences were deposited in the NCBI Sequence Read Archive database (https://www.ncbi.nlm.nih.gov/) with the accession number PRJNA1032235.

## Ethics statement

The studies involving humans were approved by the Ethics Committee of Henan Provincial People’s Hospital. The studies were conducted in accordance with the local legislation and institutional requirements. Written informed consent for participation in this study was provided by the participants’ legal guardians/next of kin. Written informed consent was obtained from the individual(s), and minor(s)’ legal guardian/next of kin, for the publication of any potentially identifiable images or data included in this article.

## Author contributions

XG: Methodology, Validation, Visualization, Writing – original draft. YL: Conceptualization, Funding acquisition, Methodology, Writing – review & editing. WX: Methodology, Visualization, Writing – review & editing. GL: Data curation, Visualization, Writing – review & editing. BX: Data curation, Investigation, Writing – review & editing. YF: Writing – review & editing. ST: Writing – review & editing. WW: Writing – review & editing. HY: Conceptualization, Funding acquisition, Writing – review & editing.
