# Supplementary material for: Eukaryotes may play an important ecological role in the gut microbiome of Graves’ disease
Source: Front Immunol. 2024 Feb 22;15:1334158. doi: 10.3389/fimmu.2024.1334158 (PMC10917987; doi:10.3389/fimmu.2024.1334158)
Supplement: Supplementary file 1 [file DataSheet_1.docx]

**Supplementary Materials and methods:**

**2.1 Inclusion and exclusion criteria of participants**

The inclusion criteria were as follows: (1) all participants were of Han nationality and were born in central plains of China; (2) GD patients were diagnosed based on typical clinical presentation, the presence of hyperthyroidism, positive serum TRAb or high radioactive iodine uptake; (3) All GD patients were newly diagnosed or relapsed patients without any treatment; (4) The control group consisted of 20 healthy volunteers with no medical or family history of thyroid diseases and had normal thyroid function and negative autoantibody responses; (5) The Recovered group consisted of 19 GD patients who were relieved of typical clinical symptoms and physical signs after medication, such as hypermetabolism and thyroid enlargement *et al.*

The following exclusion criteria applied to all groups: (1) body mass index (BMI) > 27 Kg/m^2^; (2) no antibiotic use history within 3 months prior to enrolment; (3) had diabetes mellitus, malignancy or infectious diseases; (4) had other autoimmune disease, such as SLE, RA, IBD, T1DM and multiple sclerosis (MS); (5) had immunosuppressive drug; (6) had undergone gastrectomy, fundoplication, colostomy or other digestive system surgery; (7) had persistent vomiting or a suspected gastrointestinal obstruction; (8) had severe cardiovascular and cerebrovascular diseases or liver, kidney and haematopoietic system diseases; (9) had alcoholism (drinking more than 5 times in one week, more than 100 g of spirits, 250 g of rice wine or 5 bottles of beer); (10) were pregnant.

**Supplementary figures and tables:**

Table S1 sample information of all participants

| Sample | Group | Gender | age | BMI | FT3 | FT4 | TSH | TgAb | TPOAb | TRAb | IL6 | TNFα | IL17 | IL4 | IL10 |
| --- | --- | --- | --- | --- | --- | --- | --- | --- | --- | --- | --- | --- | --- | --- | --- |
| C01 | Health | Female | 30 | 22.10 | 2.39 | 4.23 | 1.20 | 0.23 | 0.63 | 0.38 | 0.99 | 6.01 | 3.99 | 2.02 | 3.80 |
| C03 | Health | Female | 30 | 19.90 | 2.46 | 3.98 | 1.91 | 0.23 | 0.89 | 0.38 | 2.38 | 5.69 | 3.52 | 3.49 | 4.02 |
| C04 | Health | Female | 29 | 20.40 | 2.29 | 3.86 | 1.58 | 0.58 | 1.40 | 0.38 | 3.05 | 5.63 | 5.05 | 1.18 | 4.45 |
| C08 | Health | Male | 27 | 23.10 | 2.66 | 4.05 | 1.63 | 0.14 | 1.29 | 0.38 | 0.99 | 6.82 | 3.37 | 0.69 | 4.47 |
| C09 | Health | Female | 35 | 21.20 | 2.75 | 4.41 | 1.73 | 0.14 | 2.40 | 0.38 | 1.46 | 5.66 | 4.31 | 3.62 | 3.98 |
| C10 | Health | Female | 27 | 19.10 | 2.54 | 4.12 | 1.48 | 0.23 | 0.69 | 0.38 | 0.74 | 5.42 | 4.78 | 1.77 | 4.04 |
| C12 | Health | Female | 33 | 20.90 | 2.52 | 3.94 | 2.39 | 0.23 | 0.32 | 0.38 | 1.87 | 4.77 | 5.23 | 1.09 | 3.44 |
| C14 | Health | Female | 29 | 21.60 | 2.41 | 4.14 | 1.68 | 0.23 | 0.58 | 0.38 | 3.67 | 5.80 | 3.78 | 1.47 | 4.04 |
| C15 | Health | Female | 29 | 22.50 | 2.49 | 4.18 | 2.16 | 0.14 | 0.68 | 0.38 | 1.99 | 6.85 | 5.01 | 3.05 | 3.90 |
| C16 | Health | Female | 35 | 23.30 | 2.70 | 4.33 | 1.45 | 0.14 | 0.70 | 0.72 | 1.54 | 6.27 | 3.37 | 3.14 | 4.22 |
| C18 | Health | Female | 43 | 22.00 | 2.55 | 4.30 | 2.42 | 0.23 | 1.76 | 0.38 | 1.68 | 4.70 | 5.07 | 2.38 | 4.04 |
| C20 | Health | Female | 49 | 23.30 | 2.28 | 4.31 | 2.39 | 0.26 | 1.31 | 0.38 | 1.61 | 5.26 | 4.73 | 1.18 | 4.39 |
| C22 | Health | Female | 23 | 18.60 | 2.70 | 4.27 | 1.82 | 0.49 | 0.92 | 0.56 | 4.22 | 5.92 | 4.85 | 4.59 | 5.02 |
| C27 | Health | Male | 26 | 26.70 | 2.82 | 4.54 | 1.20 | 0.23 | 0.86 | 0.82 | 1.41 | 6.91 | 3.43 | 1.00 | 3.29 |
| C41 | Health | Female | 30 | 19.90 | 2.69 | 3.98 | 1.37 | 0.26 | 0.69 | 0.38 | 4.27 | 6.45 | 3.28 | 2.42 | 2.67 |
| C42 | Health | Female | 42 | 22.60 | 2.53 | 3.95 | 1.31 | 0.23 | 0.77 | 0.57 | 1.46 | 6.17 | 3.42 | 2.89 | 2.57 |
| C43 | Health | Male | 31 | 24.70 | 2.80 | 4.54 | 1.47 | 0.23 | 1.08 | 0.38 | 1.68 | 5.81 | 3.82 | 1.26 | 2.57 |
| C44 | Health | Female | 28 | 19.20 | 2.53 | 3.97 | 1.20 | 0.23 | 0.37 | 0.52 | 1.09 | 5.05 | 3.31 | 2.84 | 3.13 |
| C45 | Health | Male | 33 | 27.00 | 2.65 | 4.06 | 1.94 | 0.23 | 2.87 | 0.51 | 4.32 | 6.58 | 3.56 | 0.44 | 2.57 |
| C46 | Health | Female | 18 | NA | 2.43 | 4.17 | 2.24 | 0.26 | 0.49 | NA | 1.71 | 4.43 | 2.23 | 1.57 | 1.84 |
| G02V2 | Recover | Female | 21 | 18.29 | 3.03 | 4.64 | 0.01 | 8.68 | 9.93 | 2.40 | 1.96 | 6.80 | 3.24 | 1.94 | 3.08 |
| G04V2 | Recover | Female | 48 | 22.04 | 3.64 | 4.94 | 0.01 | 0.58 | 5.35 | 3.00 | 1.10 | 6.26 | 2.17 | 1.59 | 3.83 |
| G07V2 | Recover | Female | 25 | 21.78 | 2.93 | 4.85 | 0.01 | 0.26 | 7.74 | 1.74 | 3.08 | 5.57 | 4.05 | 1.27 | 3.06 |
| G101 | Disease | Female | 43 | 23.40 | 3.34 | 4.86 | 0.01 | 5.69 | 10.00 | 4.11 | 1.57 | 5.16 | 3.16 | 1.40 | 2.35 |
| G102 | Disease | Female | 34 | 19.50 | 5.21 | 6.66 | 0.01 | 7.64 | 9.05 | 5.28 | 1.84 | 5.86 | 4.49 | 2.23 | 4.49 |
| G112 | Disease | Female | 33 | 18.80 | 4.72 | 6.59 | 0.01 | 2.98 | 7.10 | 2.08 | NA | NA | NA | NA | NA |
| G121 | Disease | Female | 29 | 22.86 | 2.00 | 4.49 | 0.01 | 2.20 | 9.96 | 2.71 | 1.59 | 4.82 | 4.94 | 1.69 | 5.14 |
| G14V2 | Recover | Female | 21 | 19.48 | 2.74 | 4.24 | 0.01 | 0.26 | 7.58 | 4.98 | 0.72 | 7.05 | 2.60 | 1.86 | 3.91 |
| G16V2 | Recover | Female | 43 | 20.29 | 3.50 | 4.94 | 0.01 | 2.32 | 6.71 | 2.65 | 2.37 | 5.89 | 3.79 | 0.48 | 3.69 |
| G19V1 | Disease | Female | 27 | 17.51 | 5.34 | 6.66 | 0.01 | 7.57 | 8.16 | 3.27 | 1.71 | 6.37 | 3.13 | 1.38 | 3.61 |
| G20V1 | Disease | Female | 29 | 22.58 | 5.07 | 6.60 | 0.01 | 7.42 | 9.68 | 2.57 | 1.47 | 6.32 | 4.49 | 1.59 | 3.08 |
| G24V2 | Recover | Female | 36 | 20.70 | 3.61 | 4.83 | 0.01 | 0.14 | 1.92 | 2.17 | 3.39 | 6.26 | 3.11 | 3.19 | 4.44 |
| G26V2 | Recover | Female | 26 | 25.39 | 3.41 | 5.30 | 0.01 | 8.61 | 9.93 | 2.48 | 1.76 | 8.14 | 3.90 | 2.50 | 4.10 |
| G34V2 | Recover | Female | 20 | 17.04 | 2.95 | 4.47 | 0.01 | 9.54 | 10.02 | 2.57 | 2.53 | 6.04 | 3.29 | 1.38 | 3.67 |
| G36V1 | Disease | Male | 24 | 23.29 | 5.26 | 6.49 | 0.01 | 0.14 | 2.86 | 5.36 | 2.47 | 6.29 | 5.81 | 0.85 | 3.98 |
| G50V1 | Disease | Female | 32 | 23.87 | 4.79 | 5.79 | 0.01 | 2.98 | 8.76 | 3.14 | 2.01 | 6.90 | 3.57 | 1.49 | 3.76 |
| G53V1 | Disease | Female | 33 | 22.66 | 5.24 | 6.66 | 0.01 | 6.12 | 3.60 | 3.01 | 1.18 | 4.96 | 4.12 | 4.13 | 3.98 |
| G66V1 | Disease | Female | 28 | 16.10 | 5.19 | 6.66 | 0.01 | 7.93 | 9.11 | 4.64 | 3.51 | 7.27 | 3.92 | 2.31 | 2.63 |
| G72V1 | Disease | Female | 24 | 18.80 | 4.28 | 5.58 | 0.01 | 7.28 | 10.00 | 3.30 | 1.33 | 6.48 | 3.50 | 2.61 | 3.86 |
| G73V1 | Disease | Female | 38 | 25.39 | 4.93 | 6.35 | 0.01 | 1.89 | 3.13 | 3.92 | 2.48 | 6.23 | 4.53 | 1.77 | 4.12 |
| G75V1 | Disease | Female | 48 | 19.53 | 5.48 | 6.66 | 0.01 | 7.14 | 10.00 | 4.34 | 0.53 | 6.76 | 3.02 | 2.84 | 1.22 |
| G77V1 | Disease | Female | 31 | 20.00 | 4.00 | 5.41 | 0.01 | 6.87 | 9.08 | 4.31 | 2.17 | 5.85 | 4.03 | 1.72 | 2.23 |
| G81V1 | Disease | Male | 20 | 21.00 | 4.96 | 5.98 | 0.01 | 2.54 | 5.33 | 5.36 | 1.71 | 6.46 | 3.05 | 2.11 | 3.84 |
| G87 | Disease | Male | 21 | 17.90 | 5.32 | 6.59 | 0.01 | 1.00 | 10.00 | 5.36 | 3.13 | 5.46 | 4.51 | 2.73 | 2.67 |
| G89 | Disease | Female | 30 | 22.40 | 4.20 | 5.63 | 0.01 | 1.20 | 0.77 | 2.03 | 2.95 | 5.98 | 3.02 | 1.93 | 4.72 |
| G94 | Disease | Male | 21 | 21.60 | 5.07 | 6.43 | 0.01 | 8.58 | 9.71 | 3.30 | 2.26 | 6.73 | 2.72 | 0.44 | 3.29 |
| G95 | Disease | Female | 32 | 20.50 | 5.37 | 6.66 | 0.01 | 10.90 | 10.00 | 2.81 | 2.17 | 6.02 | 4.34 | 2.07 | 3.88 |
| GD120 | Recover | Female | 22 | 17.99 | 2.88 | 4.43 | 0.01 | 5.13 | 9.59 | 3.82 | 2.26 | 6.54 | 4.61 | 1.38 | 3.55 |
| GD122 | Recover | Female | 29 | 20.34 | 2.77 | 4.41 | 0.04 | 10.99 | 9.96 | 5.23 | 2.01 | 7.22 | 3.26 | 2.90 | 3.43 |
| GD123 | Recover | Female | 31 | 25.00 | 2.90 | 4.36 | 0.01 | 10.72 | 9.75 | 3.08 | 2.94 | 5.81 | 5.28 | 0.48 | 6.01 |
| GD124 | Recover | Female | 18 | 21.67 | 4.28 | 3.80 | 0.01 | 10.35 | 9.96 | 5.36 | 1.18 | 6.97 | 0.89 | 2.28 | 4.68 |
| GD125 | Recover | Female | 32 | 19.03 | 4.04 | 5.30 | 0.01 | 9.33 | 9.96 | 2.54 | 2.76 | 4.69 | 3.68 | 1.49 | 4.57 |
| GD127 | Recover | Female | 27 | 25.71 | 4.68 | 5.90 | 0.01 | 7.83 | 9.53 | 4.85 | 1.92 | 6.58 | 2.72 | 3.72 | 4.17 |
| GD130 | Recover | Female | 21 | 18.00 | 3.20 | 4.54 | 0.04 | 10.14 | 9.82 | 4.91 | 2.92 | 5.31 | 2.21 | 1.69 | 3.67 |
| GD131 | Recover | Female | 19 | 20.52 | 3.59 | 4.44 | 0.01 | 1.43 | 1.34 | 4.34 | 1.47 | 5.95 | 4.71 | 1.94 | 3.98 |
| GD132 | Recover | Male | 33 | 26.83 | 3.22 | 4.37 | 0.19 | 0.23 | 0.51 | 2.60 | 2.87 | 4.14 | 4.20 | 0.67 | 4.79 |
| GD137 | Recover | Female | NA | NA | NA | NA | NA | NA | NA | NA | NA | NA | NA | NA | NA |
| GD138 | Recover | Female | 38 | 26.37 | 2.82 | 4.39 | 0.01 | 0.23 | 4.90 | 3.13 | 1.53 | 6.99 | 2.44 | 1.38 | 3.43 |
| GD67 | Disease | Female | 17 | 18.17 | 4.97 | 6.30 | 0.01 | 6.57 | 9.32 | 3.66 | 2.07 | 7.79 | 3.99 | 3.59 | 3.55 |


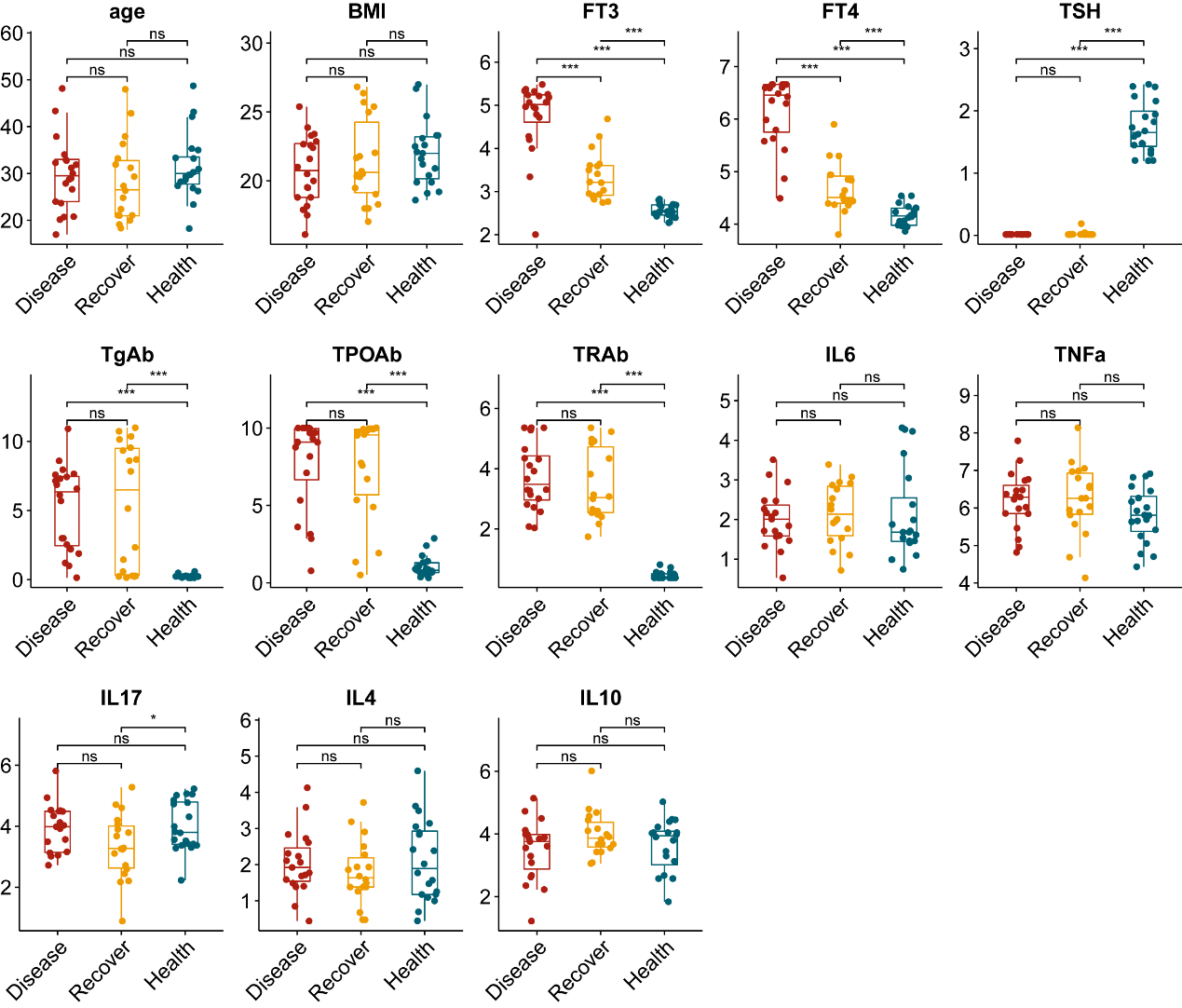


Fig. S1 age, BMI and the concentrations of physiological indexes among groups. Asterisk indicates significant difference between two groups (Wilcoxon Signed-Rank test, *p ≤ 0.05, ***p ≤ 0.001)


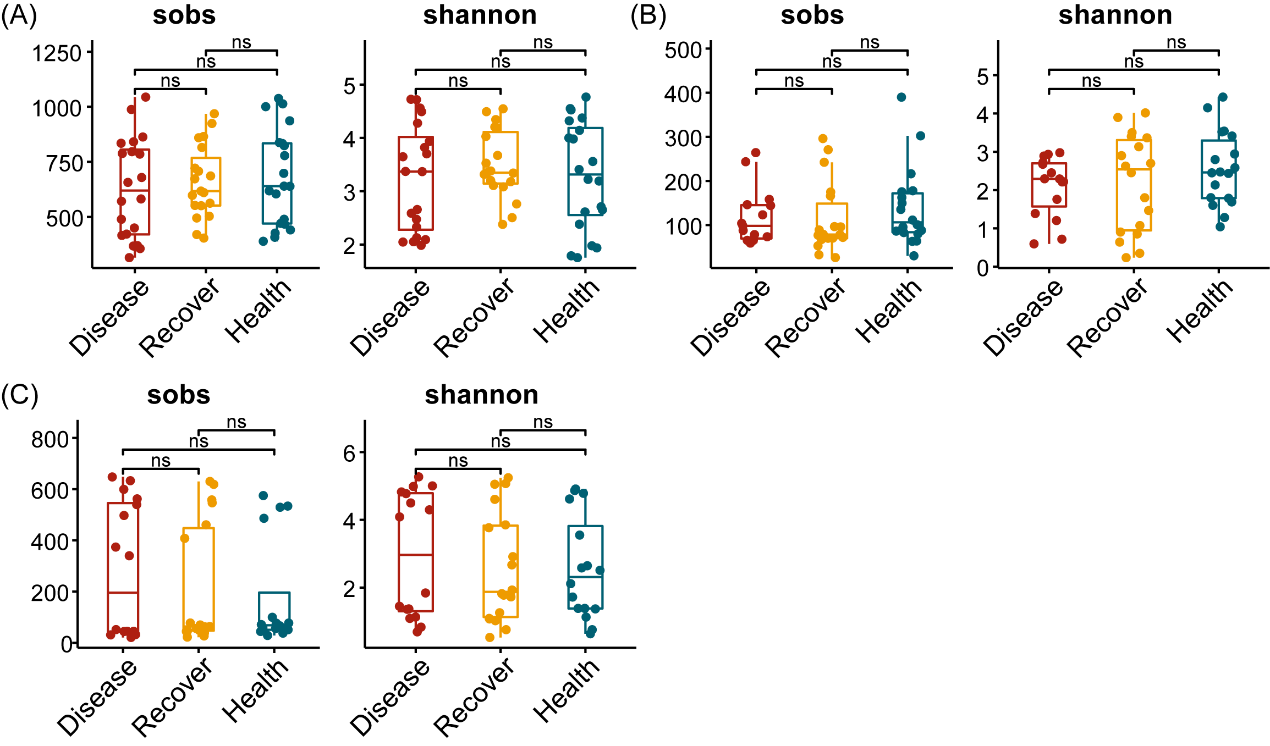


Fig. S2 Microbial richness (sobs) and evenness (shannon) among three groups. (a) Diversity in bacterial communities. (b) Diversity in fungal communities. (c) Diversity in bacterial communities. “ns” indicated there is no significant difference between two groups (Wilcoxon Signed-Rank test, p > 0.05)


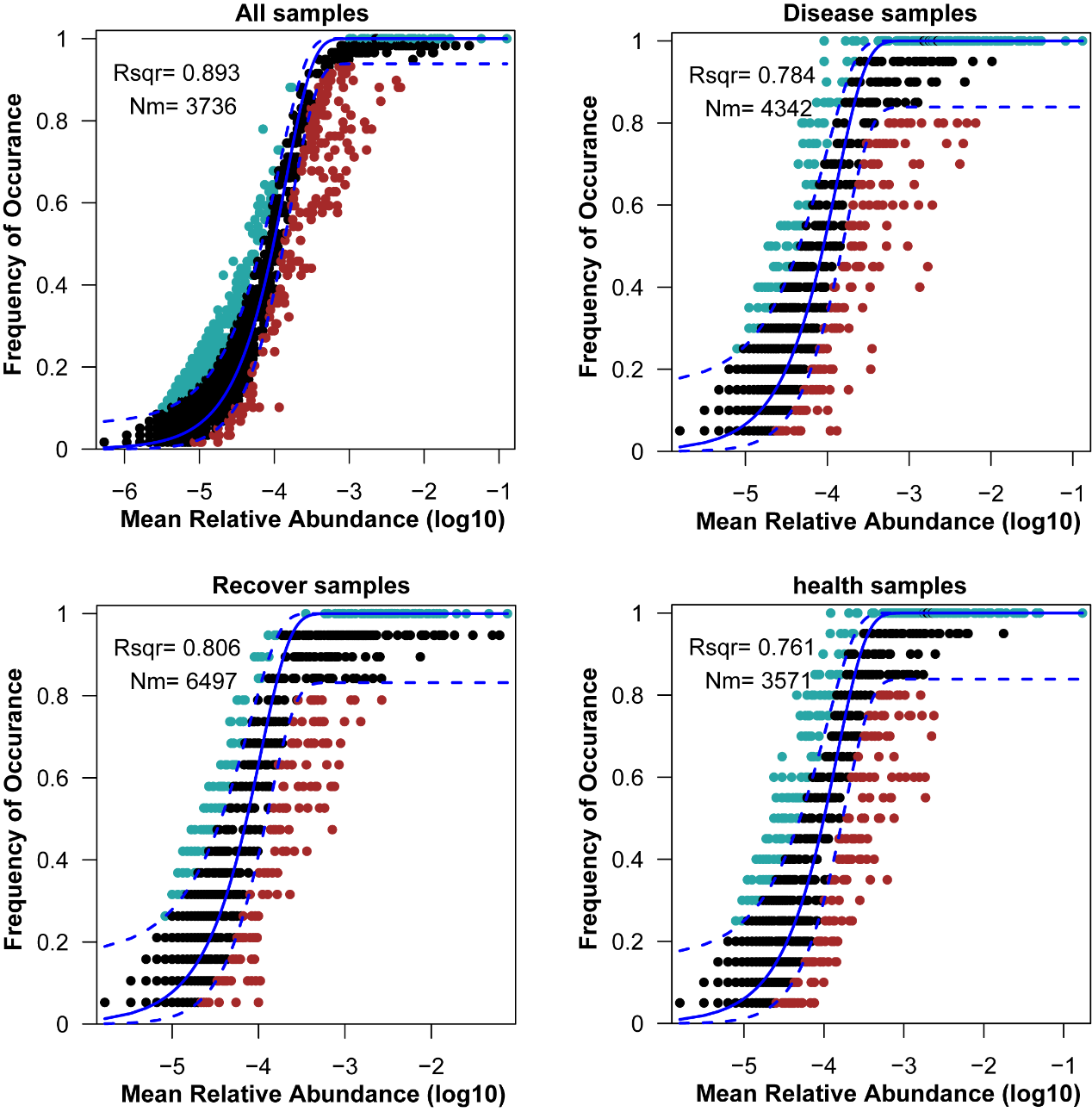


Fig. S3 Fit of the neutral community model (NCM) for bacterial community assembly. The predicted occurrence frequencies for Disease, Recover, Health and all representing communities from Disease, Recover, Health, respectively. The solid blue lines indicate the best fit to the NCM, and the dashed blue lines represent 95% confidence intervals around the model prediction. zOTUs that occur more or less frequently than predicted by the NCM are shown in different colors. Nm indicates the metacommunity size times immigration, R2 indicates the fit to this model


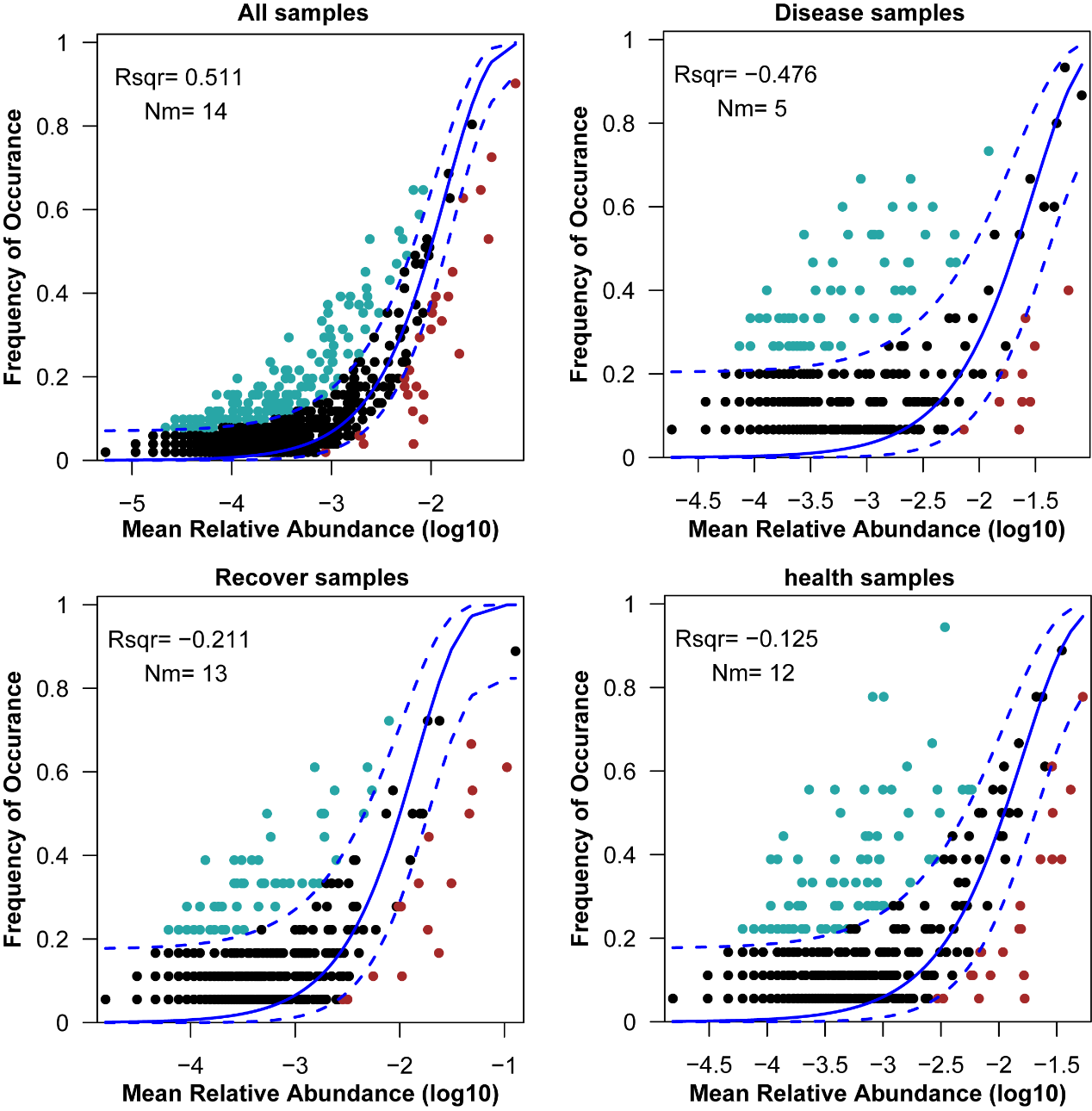


Fig. S4 Fit of the neutral community model (NCM) for fungal community assembly. The predicted occurrence frequencies for Disease, Recover, Health and all representing communities from Disease, Recover, Health, respectively. The solid blue lines indicate the best fit to the NCM, and the dashed blue lines represent 95% confidence intervals around the model prediction. zOTUs that occur more or less frequently than predicted by the NCM are shown in different colors. Nm indicates the metacommunity size times immigration, R2 indicates the fit to this model.


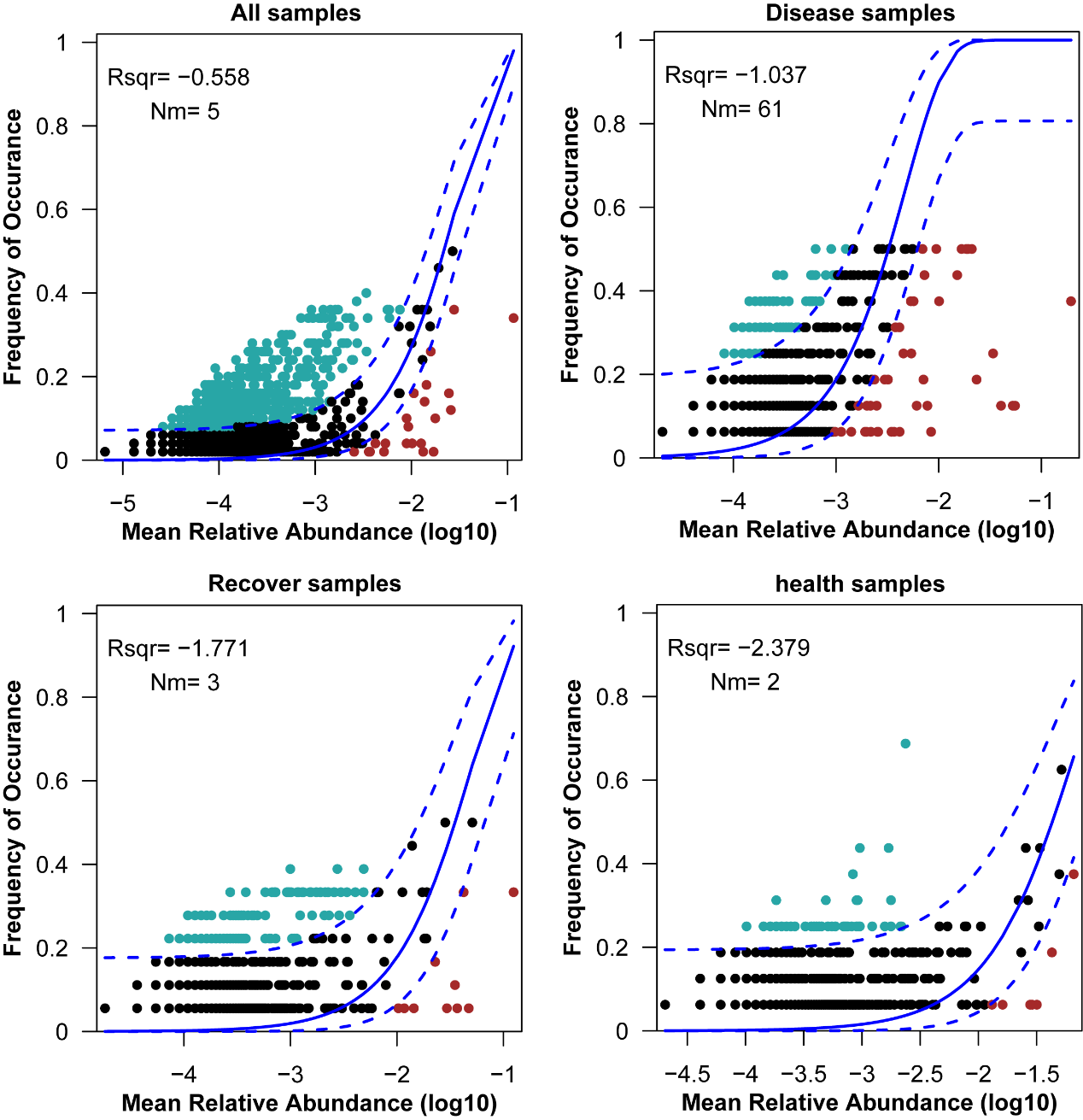


Fig. S5 Fit of the neutral community model (NCM) for protistan community assembly. The predicted occurrence frequencies for Disease, Recover, Health and all representing communities from Disease, Recover, Health, respectively. The solid blue lines indicate the best fit to the NCM, and the dashed blue lines represent 95% confidence intervals around the model prediction. zOTUs that occur more or less frequently than predicted by the NCM are shown in different colors. Nm indicates the metacommunity size times immigration, R2 indicates the fit to this model


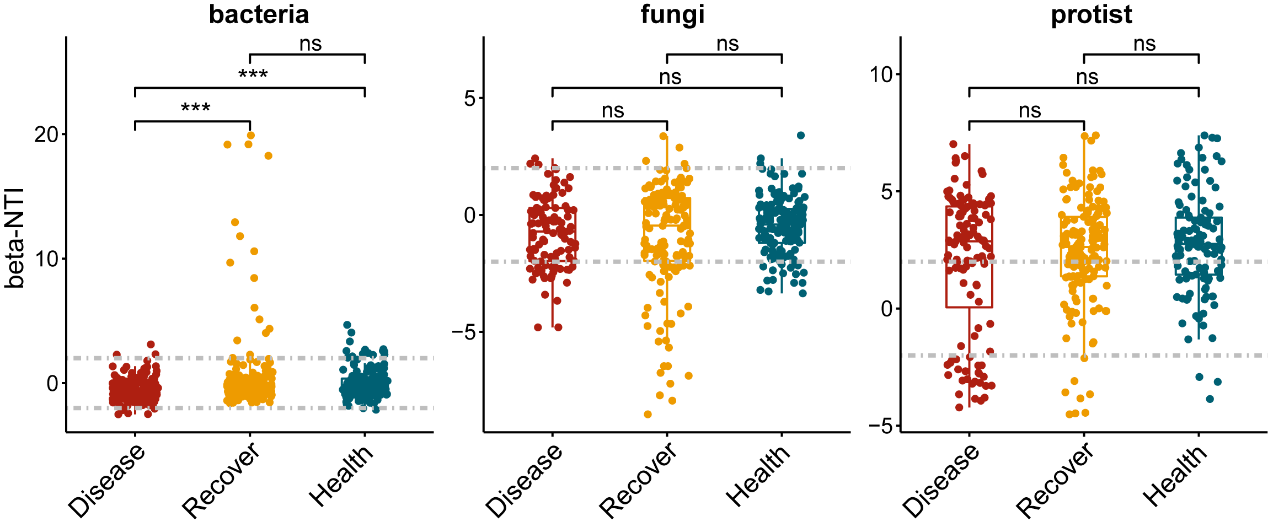


Fig. S6 Contributions of deterministic and stochastic processes on community assembly in the sampling data. Asterisk indicates significant difference between two groups (Wilcoxon Signed-Rank test, ***p ≤ 0.001)


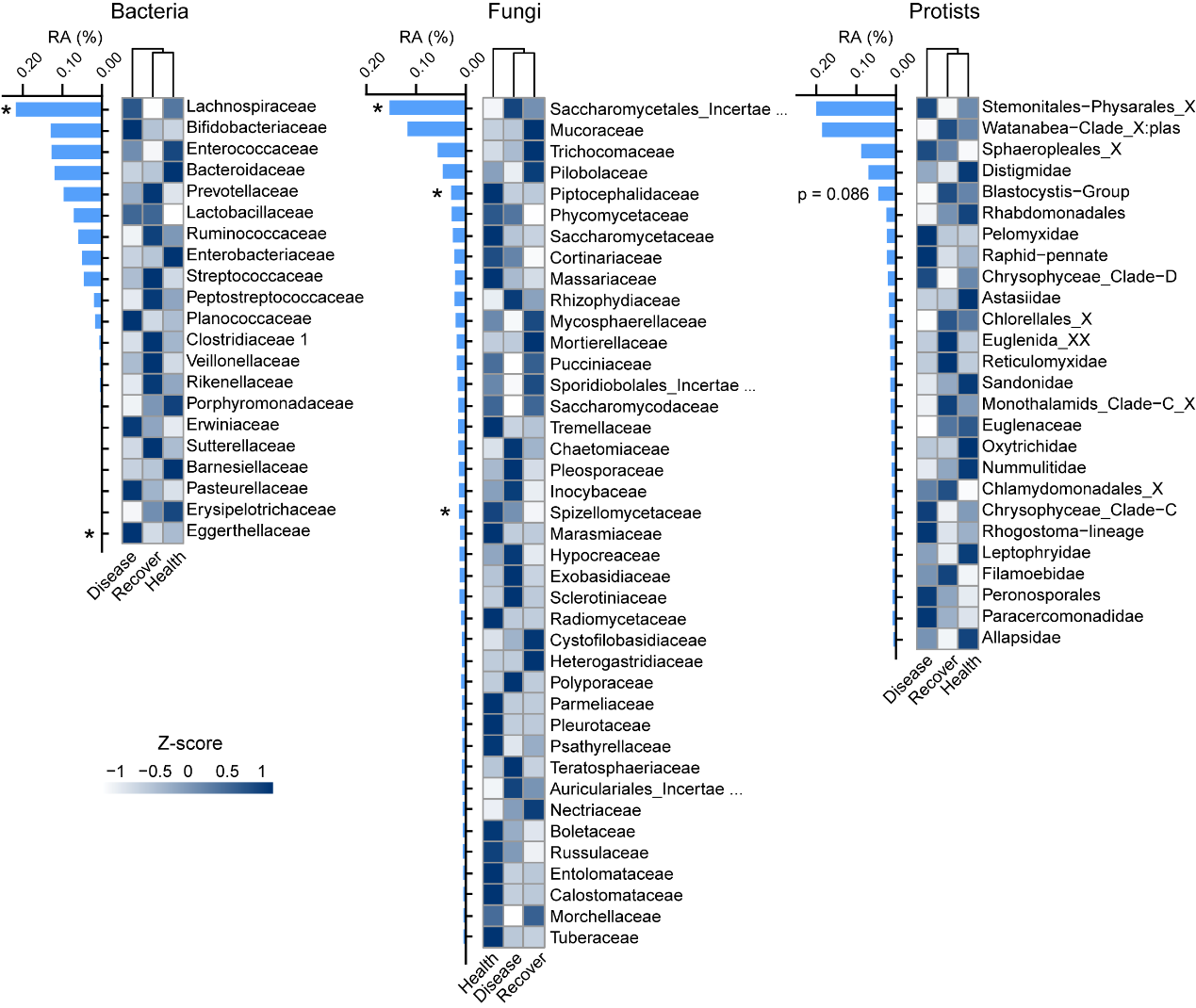


Fig. S7 Relative abundance of bacterial, fungal and protistan taxonomy at family level (RA>0.1%) of all treatments. Asterisk indicates significant difference among all groups (Kruskal-Wallis test, *p ≤ 0.05)


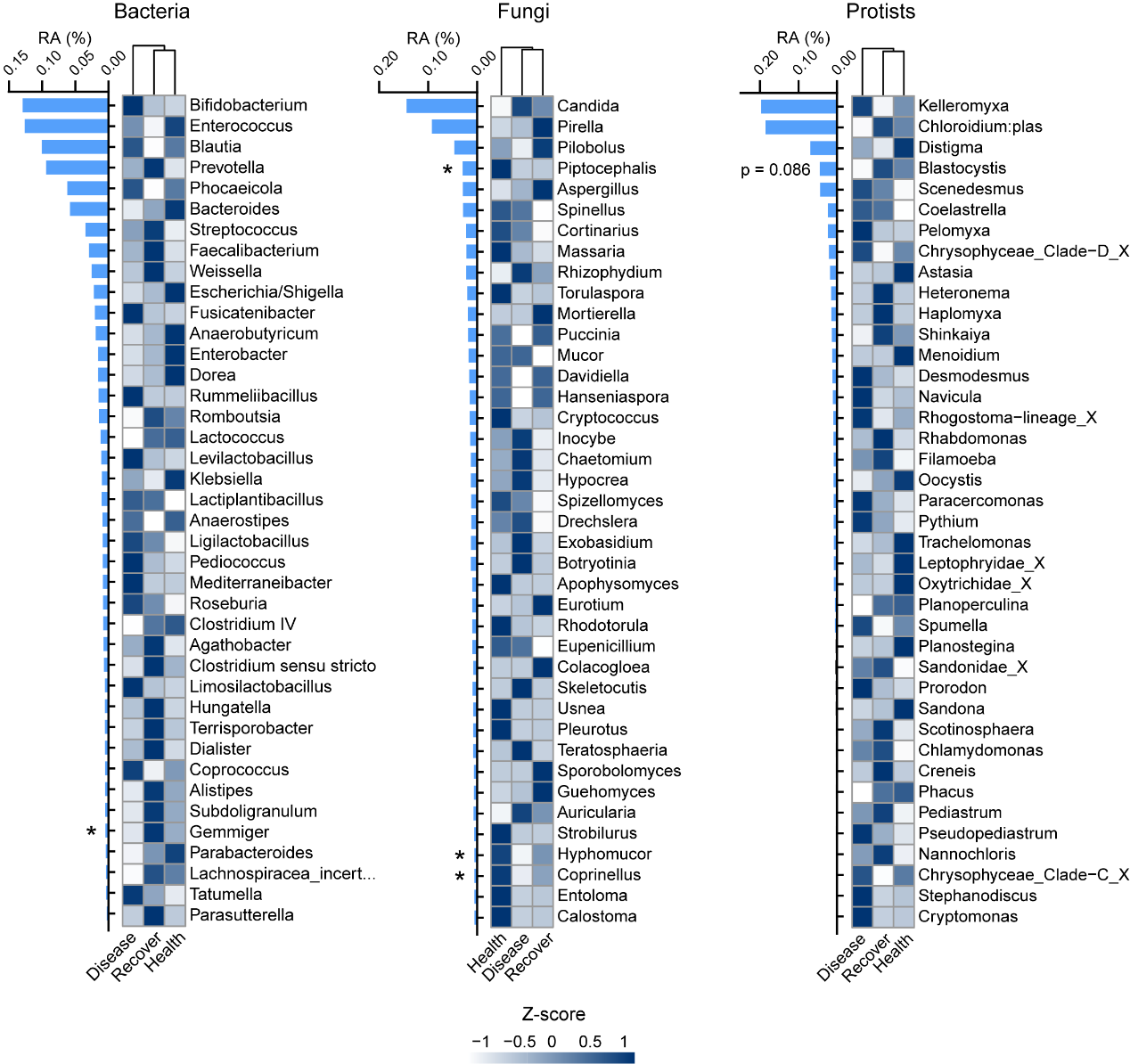


Fig. S8 Relative abundance of bacterial, fungal and protistan taxonomy at genus level (40 most abundant genus for each kingdom) of all treatments. Asterisk indicates significant difference among all groups (Kruskal-Wallis test, *p ≤ 0.05)

**Reference:**

1. Stegen JC, Lin X, Fredrickson JK, Chen X, Kennedy DW, Murray CJ, Rockhold ML, Konopka A. Quantifying community assembly processes and identifying features that impose them. *The ISME Journal 2013 7:11* (2013) 7:2069–2079. doi: 10.1038/ismej.2013.93
